# Superficial Dsg2 Expression Limits Epidermal Blister Formation Mediated by Pemphigus Foliaceus Antibodies and Exfoliative Toxins

**DOI:** 10.1155/2010/410278

**Published:** 2010-06-09

**Authors:** Donna Brennan, Ying Hu, Walid Medhat, Alicia Dowling, Mỹ G. Mahoney

**Affiliations:** Department of Dermatology and Cutaneous Biology, Jefferson Medical College, Thomas Jefferson University, Philadelphia, PA 19107, USA

## Abstract

Cell-cell adhesion mediated by desmosomes is crucial for maintaining proper epidermal structure and function, as evidenced by several severe and potentially fatal skin disorders involving impairment of desmosomal proteins. Pemphigus foliaceus (PF) and staphylococcal scalded skin syndrome (SSSS) are subcorneal blistering diseases resulting from loss of function of the desmosomal cadherin, desmoglein 1 (Dsg1). To further study the pathomechanism of these diseases and to assess the adhesive properties of Dsg2, we employed a recently established transgenic (Tg) mouse model expressing Dsg2 in the superficial epidermis. Neonatal Tg and wild type (WT) mice were injected with purified ETA or PF Ig. We showed that ectopic expression of Dsg2 reduced the extent of blister formation in response to both ETA and PF Ig. In response to PF Ig, we observed either a dramatic loss or a reorganization of Dsg1-*α*, Dsg1-*β*, and, to a lesser extent, Dsg1-*γ*, in WT mice. The Inv-Dsg2 Tg mice showed enhanced retention of Dsg1 at the cell-cell border. Collectively, our data support the role for Dsg2 in cell adhesion and suggest that ectopic superficial expression of Dsg2 can increase membrane preservation of Dsg1 and limit epidermal blister formation mediated by PF antibodies and exfoliative toxins.

## 1. Introduction

Desmogleins and desmocollins are the transmembrane adhesive components of intercellular junctions known as desmosomes. Four distinct desmogleins (Dsg 1-4) have been reported with unique expression profiles dependent on the tissue type and differentiation state [1, 2]. Unlike Dsg1 and Dsg3, whose expression is restricted to complex stratified epithelia, Dsg2 and Dsg4 are expressed in a wide range of other cell types. In stratified epithelia, such as the human epidermis, Dsg2 is expressed at low levels and is restricted to the proliferative basal cell layer while Dsg3 expression extends from the basal into the spinous cell layers. Conversely, Dsg1 and Dsg4 expression is driven by cell differentiation. Dsg1 is expressed from the immediate suprabasal layer up, with marked higher abundance in the differentiated granular cell layers. Dsg4 expression is restricted to the highly differentiated upper cell layers. 

It is well established that desmosomal cadherins play a significant role in cell adhesion and tissue integrity, as they have been identified as the target proteins in several autoimmune, infectious, and inherited skin/hair fragility diseases [3]. Pemphigus is a devastating and debilitating autoimmune skin disease [4]. This disorder is generally characterized by the production of pathogenic antibodies targeting different components of cell-cell adhesion, in particular, Dsg1 and Dsg3. The autoantibodies commonly cause cell-cell disadhesion (acantholysis), as well as blisters of the skin and mucous membranes. In pemphigus foliaceus (PF), patients develop pathogenic autoantibodies that target Dsg1 and promote cell-cell disadhesion in the superficial epidermis, where Dsg1 is highly expressed, but which lacks Dsg3 and Dsg2. Passive transfers of purified PF Ig into newborn mice produce blisters similar to those observed in PF patients [5, 6]. Interestingly, in the toxin-mediated disease bullous impetigo (and its generalized form staphylococcal scalded skin syndrome (SSSS)), the bacterium *S. aureus* produces exfoliative toxins (glutamic-specific serine proteases) that cleave Dsg1 between extracellular domains 3 and 4 [7]. Reminiscent of PF, SSSS patients develop superficial skin blisters [8]. Furthermore, newborn mice treated with exfoliative toxins develop skin blisters similar to those observed with the passive transfer of PF Ig. Both pathogenic pemphigus autoantibodies and exfoliative toxins target specific conformational epitopes found within the N-terminal extracellular domains of desmogleins [9]. These domains are believed to play an important role in cadherin-cadherin interaction, thereby alluding to the importance of desmogleins in controlling intercellular adhesion and in maintaining the structural stability and integrity of the epidermis [10–13]. 

To assess the ability of Dsg2 to enhance cell adhesion and to test the hypothesis that Dsg2 expression in the suprabasal epidermis can limit PF/ETA blister formation by increasing keratinocyte adhesion, we employed a transgenic mouse model expressing Dsg2 in the superficial epidermis under the involucrin promoter (Inv-Dsg2 Tg) [14]. We selected Dsg2 since it is not a pemphigus antigen [15] and does not appear to have the consensus sequence required for cleavage by exfoliative toxins. We subjected the Tg mice and their WT littermates to ETA and PF Ig treatments and assessed the extent of skin blister formation. These experiments allowed us to compare the relative intensity of the ETA- and PF-induced blister formation in the presence or absence of Dsg2 in the superficial epidermis. The results obtained here provide some insights into the pathomechanisms of diseases targeting Dsg1. 

## 2. Results

### 2.1. Expression of Dsg2 in the Superficial Epidermis of Inv-Dsg2 Tg Mice

**Figure 1:**
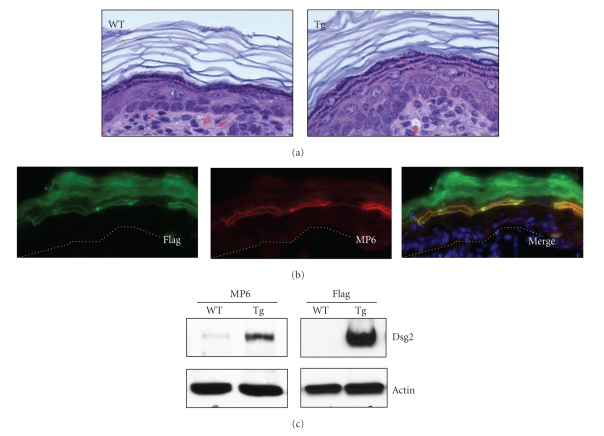
Suprabasal expression of Dsg2-Flag in newborn Inv-Dsg2 transgenic mice. (a) Histology showing slight epidermal hyperplasia in a newborn Inv-Dsg2 Tg overexpressing Dsg2 in the superficial epidermis under the involucrin promoter, but not in WT mice. (b) Immunostaining of newborn Tg skin with Flag (green) and Dsg2-specific MP6 (red) antibodies showing expression of Dsg2-Flag in the differentiated cell layers of the transgenic epidermis. (c) Immunoblot of WT and Tg skin with MP6 and Flag antibodies, showing Dsg2-Flag in the Tg but not WT skin. Actin was used for equal loading.

As previously described in detail, we generated Tg mice expressing Dsg2 in the superficial epidermis, under the control of the involucrin promoter [14]. Newborn Tg mice appeared normal, with no gross abnormalities of the skin or hair. Examination of the skin by histology revealed minor epidermal hyperplasia in newborn Tg mice compared to WT littermates (Figure 1(a)). We assessed the expression of the Dsg2-Flag transgene in skin from newborn Tg mice by immunostaining; antibodies against Flag and Dsg2 (MP6) (Figure 1(b)) showed expression of Dsg2-Flag in the superficial cell layers. In keeping with the literature, we observed some negligible expression of endogenous Dsg2 in the basal cell layer. 

To confirm the immunoblotting results, we extracted total skin protein from WT and Tg skin in RIPA buffer and resolved it with SDS-PAGE. Immunoblotting with the MP6 and Flag antibodies detected bands of approximately 160 kDa in Tg skin lysates (Figure 1(c)). MP6, but not Flag, antibody picked up a weak signal for a similar sized band in the WT skin, which is indicative of low levels of endogenous Dsg2 in the newborn mouse skin. In summary, we generated transgenic mice expressing Dsg2 in the superficial epidermis of newborn mice.

### 2.2. Dsg2 Protects Skin from ETA-Mediated Blister Formation

**Figure 2:**
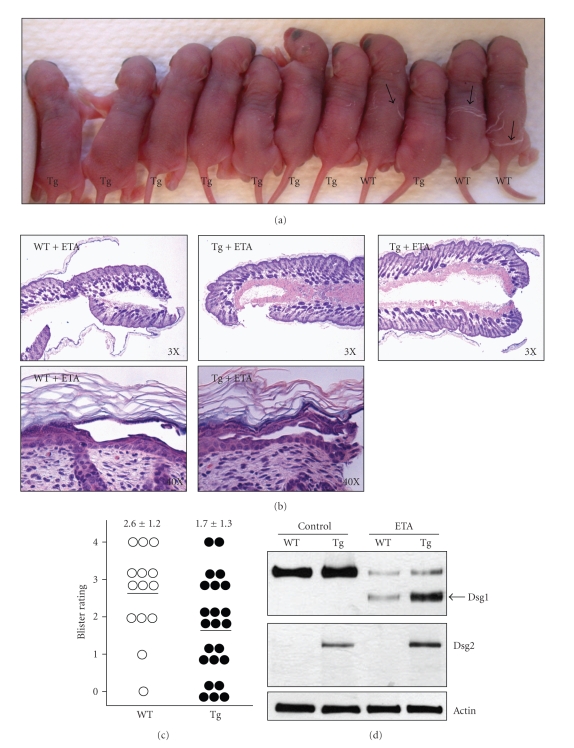
Superficial expression of Dsg2 offers protection from ETA-induced blister formation. (a) Newborn WT and Inv-Dsg2 Tg mice were injected subcutaneously with 0.5 *μ*g ETA in PBS. Visible blisters were observed in WT but not Tg mice, 6–8 hours after ETA treatment. (b) Mice were sacrificed, and their skin was processed for histological analysis, revealing slightly more extensive blisters in the WT mice (*n* = 14), as compared to Tg mice (*n* = 23), in response to ETA. Top panels show 3X magnification, and lower panels show 40X magnification of the site of blister formation. (c) The extent of blistering was graded based on the following semiquantitative scale: 0: no blisters, 1+: minor blisters at the edge; 2+: localized blisters <50%; 3+: extensive blisters >50%; and 4+: very extensive blisters >75%. Each dot represents one mouse. The average blister scores were 2.6 ± 1.2 for WT and 1.7 ± 1.3 for Tg. These values were statistically significant, *P* ≤  .038260921 (2-tailed unequal variance) or ≤  .01913046 (1-tailed unequal variance). (d) The back skin biopsies were homogenized in Laemmli buffer, proteins were resolved with SDS-PAGE and immunoblotted for Dsg1 (27B2), Dsg2-Flag (Flag), and Actin (for equal loading). Western blotting demonstrates that ETA cleaved Dsg1 (arrowhead), but not Dsg2. *Note*. antibody 27B2 was raised against the cytoplasmic epitope of human Dsg1, and recognizes mouse Dsg1-*a*, -*β*, and -*γ*. Thus, the full-length signal is most likely ETA-resistant Dsg1-*γ*.

It is well established that ETA cleaves Dsg1 and causes epidermal blisters in the upper layers of the epidermis, where Dsg1 is highly expressed [16]. Mice treated with purified ETA develop blisters similar to those seen in patients infected with *S. aureus*. To determine whether Dsg2 could compensate for the loss of Dsg1, we treated neonatal WT and Tg mice with purified ETA. In response to subcutaneous injection of ETA, we observed dramatic gross blisters in WT, but not Tg, mice (Figure 2(a)). However, the results from the histologic analysis were less definitive, since some Tg mice developed extensive blisters while others showed a resistance to blister formation (Figure 2(b), top panels). 

Upon further analysis, we observed that the site of blister formation in WT mice after ETA treatment was superficial and occurred in the middle of the granular cell layer (Figure 2(b), lower left panel), where Dsg1 is highly expressed. However, in the Inv-Dsg2 Tg skin treated with ETA, the site of epidermal splitting was often just beneath the granular cell layer (Figure 2(b), lower right panel). These results suggest that coexpression of Dsg2 with Dsg1 in the superficial epidermis may protect from the loss of Dsg1 by ETA although we cannot rule out the possibility that ectopic expression of Dsg2 may also impair the ETA-digestion of Dsg1 molecules. However, the latter scenario is unlikely, as ETA is enzymatically efficient, and we did not observe any significant increase in uncleaved Dsg1 in ETA-treated Tg mice (see below). 

The extent of the histologic blistering was graded on a scale from 0, for no blisters, to +4, for extensive blisters. We observed that overexpression of Dsg2 provided enhanced protection against blister formation in response to ETA (Figure 2(c)). To confirm that ETA cleaves mouse Dsg1 when injected into newborn WT and Tg mice, skin biopsies were taken after ETA treatment and the total cellular proteins were extracted in Laemmli buffer. Western blotting showed that ETA cleaves Dsg1, resulting in a smaller fragment approximately 113 kDa in both WT and Inv-Dsg2 Tg mouse skin (arrow, Figure 2(d)) [8, 16]. We previously showed that antibody 27B2 recognizes an epitope present in the cytoplasmic domain of all mouse Dsg1 (*α*, *β*, and *γ*) isoforms [17]. In that report, we also demonstrated that ETA cleaves only Dsg1-*α* and -*β*. Thus, the remaining full-length Dsg1 fragment left after ETA-treatment observed here is most likely Dsg1-*γ*, but may also show inefficient Dsg1-*α* or -*β* cleavage. Interestingly, we observed a slight increase in Dsg1 in the ETA treated Tg skin (Figure 2(d)). This increase in Dsg1 was reflected in a more intense 113 kDa band in response to ETA cleavage. Finally, we showed here that ETA did not cleave Dsg2. 

**Figure 3:**
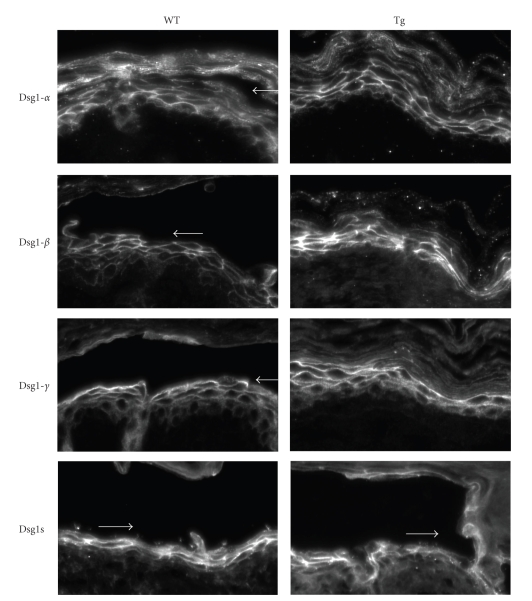
Dsg1 is maintained at the cell-cell border in ETA-treated epidermis. Formalin-fixed paraffin-embedded skin sections from newborn WT and Tg mice treated with ETA, as described in Figure 2, were immunostained with antibodies AP61, AP498, Ab15, and 27B2. Antibodies AP61, AP498, and Ab15 were raised against the extracellular domain of Dsg1-*α*, Dsg1-*β*, and Dsg1-*γ*, respectively [17]. Immunofluorescence shows undisturbed cell-cell border staining of all Dsg1 isoforms in both WT and Tg skins treated with ETA. DAPI (blue) was used as a nuclear stain. Similar results were observed in skin treated with ETA for 18 hours. Arrows demarcate site of blister cleavage.

To assess whether ectopic expression of Dsg2 in the Tg skin had an effect on Dsg1 in response to ETA, we performed immunofluorescence staining for Dsg1. We determined the expression pattern of Dsg1-*α*, -*β*, and -*γ* using antibodies specific against each Dsg1 isoform (Figure 3). These antibodies were generated against either synthetic peptides or recombinant proteins localized within the extracellular domain of Dsg1 and would thus detect the full-length, membrane-spanning product after ETA digestion [17]. Despite the extensive cleavage of Dsg1 by ETA, as shown by Western blotting above, we did not observe a dramatic change in the Dsg1 pattern in either WT or Tg skin. Dsg1 remained intact at the cell-cell border. To further confirm these results, we immunostained ETA-treated WT and Tg skin with antibody 27B2, which was raised against the intracellular domain of human Dsg1 and recognizes all three mouse Dsg1s. Staining with 27B2 showed cell-cell border staining of Dsg1s even at the blister sites (Figure 3). Collectively, our results demonstrate that ectopic expression of Dsg2 could partially compensate for the loss of Dsg1-mediated adhesion in response to ETA digestion. 

### 2.3. Dsg2 Protects against PF Ig-Mediated Blister Formation

**Figure 4:**
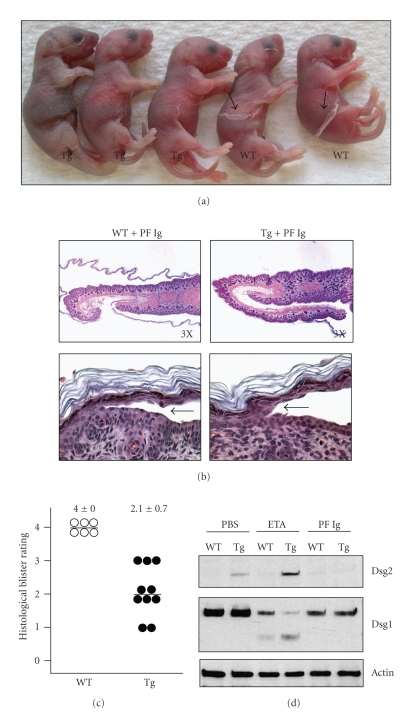
Dsg2 offers protection against PF Ig-induced acantholysis. (a) Newborn WT and Inv-Dsg2 Tg mice were injected subcutaneously with 10 mg of purified PF Ig for 18 hours. Gross blisters were more pronounced in WT mice, as compared to Tg mice. (b) Mice were sacrificed, and their skin was processed for histology, revealing more dramatic blisters in the WT (*n* = 6) than in Tg mice (*n* = 10). Top panels show 3X magnification, and lower panels show 40X magnification of the site of blister formation (arrows). (c) The extent of blistering was graded as described in Figure 2. Each dot represents one animal. The average blister scores were 4.0 ± 0.0 for WT mice and 2.1 ± 0.7 for Tg mice. These values were statistically significant, *P* ≤  .000022 (2-tailed unequal variance) or ≤  .000012 (1-tailed unequal variance). (d) Back skin biopsies of WT and Tg mice treated with PF Ig were homogenized in Laemmli buffer, the proteins were resolved with SDS-PAGE and immunoblotted for Dsg1 (27B2), Dsg2, and Actin. Western blotting demonstrates that superficial Dsg2 slightly enhances retention of Dsg1 in response to PF Ig.

Next, we wanted to assess whether ectopic expression of Dsg2 could protect against skin blister formation induced by PF pathogenic antibodies. We purified Ig from the sera of PF patients with the active disease and performed passive transfer of the PF Ig into newborn WT and Tg mice. We, and others, have shown that mice injected with normal human Ig do not develop skin blister formation [6]. In accordance with the literature, we observed extensive gross blisters in WT mice 18 hours after injection with PF Ig [6]. However, the Inv-Dsg2 Tg mice injected with PF Ig developed significantly less extensive blisters (Figure 4(a)). The severity of skin blistering was similar between gross observation and histological analysis (Figure 4(b)). Again similar to the blistering observed with ETA in Figure 2, we observed a slight downward shift in the site of blister formation in Tg, as compared to WT, skin (Figure 4(b), lower panels). The extent of histological blistering was then graded, and the results showed that Tg mice were less susceptible to blister formation in response to PF Ig (Figure 4(c)). Thus, ectopic expression of Dsg2 in the superficial epidermis rendered the Tg mice more resistant to PF Ig-induced skin blisters. 

To test the effects of PF Ig on epidermal Dsg1, skin biopsies were collected after PF Ig treatment, and the total cellular proteins were extracted in Laemmli buffer. Western blotting showed that (1) up-regulation of Dsg2 did not alter the expression level of Dsg1 in newborn mouse skin, and (2) incubation with PF Ig reduced the level of Dsg1 (Figure 4(d)). Thus, our results demonstrate that, at the Western blot level, PF Ig depletes Dsg1 and that superficial expression of Dsg2 in Tg mice did not appear to modulate the level of Dsg1 in response to PF Ig.

**Figure 5:**
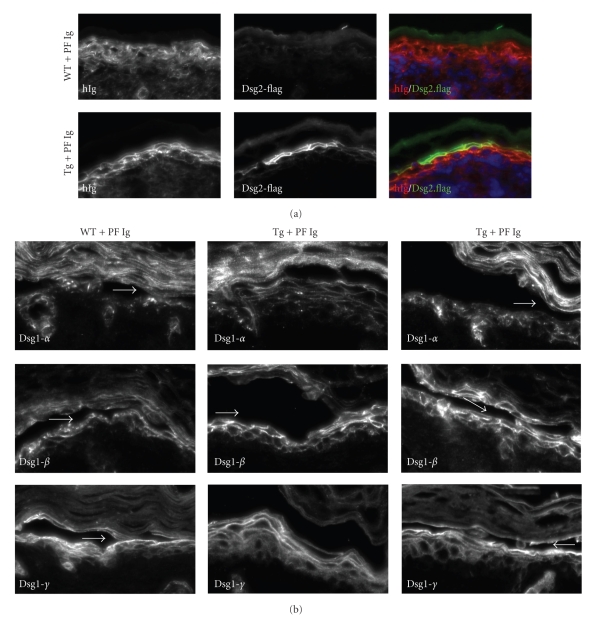
Superficial expression of Dsg2 reduces disruption of Dsg1 in response to PF Ig. Newborn WT and Dsg2 Tg mice were injected subcutaneously with PF Ig, and, 18 hours later, back skin samples were frozen in OCT or were fixed in formalin and embedded in paraffin for immunostaining. (a) Direct immunostaining for human Ig (hIg, left panels), showing localization of human antibodies to the epidermis after PF Ig passive transfer. The same tissue was immunostained with Flag antibodies, demonstrating the presence of Dsg2-Flag in the Tg, but not WT, skin (middle panels). Double labeling for the human Ig (red) and the Flag tag (green) is shown (right panels). Staining of human Ig was considerably more intact at the cell-cell border in the Tg skin, particularly in the superficial epidermis, where Dsg2 was expressed. Thus, superficial Dsg2 retains PF Ig at the cell-cell borders. (b) Lesional and nonlesional back skin sections were then immunostained with antibodies AP61, AP498, and Ab15, which were raised against the extracellular domain of Dsg1-*α*, Dsg1-*β*, and Dsg1-*γ*, respectively. DAPI (blue) was used as a nuclear stain. Arrows demarcate site of blister cleavage (lesional skin). In WT mice, treatment with PF Ig dramatically disrupted cell-cell border staining of Dsg1-*α* and Dsg1-*β* but not Dsg1-*γ* (left panels). Superficial expression of Dsg2 reduced the extent of Dsg1 perturbation (internalization and degradation). There was no significantly observable difference between lesional and non-lesional epidermis.

Next, we wanted to evaluate whether or not superficial expression of Dsg2 had an effect on Dsg1 fate and localization in response to PF Ig. We first confirmed, by direct immunostaining, the presence of human antibodies in the skin of mice treated with PF Ig (Figure 5(a), left panels). The human Ig was detected in the epidermis of both WT and Tg skin. In WT skin, staining for human antibodies was diffuse and was disrupted by some cytoplasmic staining. Interestingly, in the Tg epidermis, the staining for human antibodies was clearly at the cell-cell borders, suggesting, perhaps, the presence of more intact desmosomes (Figure 5(a), lower left panel). We also immunostained the same tissues for Dsg2-Flag to demonstrate that Tg, but not WT, mice expressed the Dsg2-Flag transgene (Figure 5(a), middle panels). Double labeling (Figure 5(a), right panels) showed colocalization (yellow) of Dsg2 (green) and human Ig (red) at the cell-cell border in the superficial epidermis (lower right panel).

Next, we assessed the expression and localization of Dsg1 (*α*, *β*, and *γ*) by indirect immunofluorescence (Figure 5(b)). In WT animals, PF Ig treatment induced extensive redistribution of Dsg1-*α* and Dsg1-*β* from a uniform cell-cell border pattern to a disrupted granular pattern, which is indicative of potential dissolution of the desmosomes (Figure 5(b), left panels). The dissolution occurred throughout the epidermis but was more extensive in the less differentiated cell layers. Intriguingly, PF Ig had a minor effect on Dsg1-*γ*, showing a slight reduction in the deep epidermis as compared to untreated control skin. In Tg mice treated with PF Ig, the presence of Dsg2 in the superficial epidermis helped retain Dsg1 at the cell-cell border (Figure 5(b), middle and right panels). The level of Dsg1 dissolution was not dramatically different between unaffected and lesional skin. In conclusion, superficial expression of Dsg2 appears to maintain the organization of Dsg1 in response to PF Ig treatment.

## 3. Discussion

In this paper, we showed that superficial expression of Dsg2 in Tg mice offers protection against skin blister formation in response to the bacterial toxin ETA and pathogenic PF antibodies. These results suggest that Dsg2 can enhance mechanical adhesion, likely contributing to this protective effect. In the case of ETA, this enhancement could counter the loss of adhesive function due to the removal of sequences in the ectodomain of Dsg1 known to be important for desmoglein-dependent adhesion [12, 13]. In the case of PF Ig, increased adhesion may compensate for possible steric hindrance of desmoglein trans-interaction induced by antibody-antigen binding [6, 18, 19]. 

Despite considerable progress in pemphigus research, there is still a controversy over the mechanism of pemphigus Ig-induced acantholysis—is it a disease of steric hindrance, cell signaling, or both? Steric hindrance of desmoglein trans-interaction induced by antibody-antigen binding was initially proposed based on evidence that one desmoglein could compensate for the loss of another [6, 18, 19]. The most compelling data supporting this hypothesis comes from a study showing that ectopic expression Dsg3 in the superficial epidermis of Tg mice [19] protects from blister formation induced by PF antibodies [20]. Furthermore, toxins such as ETA can induce PF-like skin blisters by proteolysis of Dsg1 in the absence of antibody binding [16]. In this paper, we demonstrate that ectopic expression of Dsg2 in the superficial epidermis could limit both PF Ig- and ETA-induced skin blister formation, suggesting that steric hindrance plays a role in the mechanism of pemphigus.

However, desmoglein compensation in response to pathogenic antibodies may work through mechanisms other than steric hindrance [3, 21, 22]. Indeed, in the case of PF, anti-Dsg1 antibodies can induce blister formation by triggering intracellular signaling pathways, without disrupting Dsg1-Dsg1 interactions [23]. Intracellular signaling may then promote alterations in cortical actin remodeling and may induce other changes that lead to the depletion of desmogleins from the desmosome and lead to consequent blistering. It is possible that Dsg2 counters the impact of PF Ig on these pathways, resulting in the retention of Dsg1 at the cell surface and maintenance of desmosome structure and function.

Also, suprabasal expression of Dsg2 in Tg mice offers greater protection against PF Ig, as compared to ETA (Figure 4). The difference in the response to ETA and PF Ig in transgenic mice may be due to differences in the mechanism by which ETA and PF Ig induce blistering, the mechanism by which suprabasal Dsg2 limits the blistering, or both. For instance, whereas PF antibodies disrupt and reduce cell-cell border staining of Dsg1 (Figure 5), ETA cleavage does not appear to alter the plasma membrane localization of Dsg1 despite inducing extensive skin blisters (Figure 3). It is possible that retention of this truncated Dsg1 molecule at the cell surface may attenuate Dsg2's protective effects. In addition, either alterations in the subcellular distribution of armadillo proteins or the differential impact on other signaling pathways could also account for the observed differences in Dsg2's protective effect between ETA- or PF-treated Tg mice. It has been reported that pemphigus antibodies trigger a rapid turnover and reduction of the nuclear pool of plakoglobin, thereby abolishing plakoglobin's role as a transcriptional repressor of the proto-oncogene c-Myc [24]. We recently demonstrated that Dsg2 enhances c-Myc expression [14]. In that study, we demonstrated that Dsg2 plays a role in cell signaling activation, and many signaling pathways directly involved in epithelial cell growth and survival are activated in Dsg2 Tg mice. 

In summary, we propose that desmogleins mediate cellular homeostasis through cell-cell adhesion and activation of signaling pathways; changes in desmosome structure and integrity by pemphigus antibodies or ETA can disrupt this balance. Compensating with another desmoglein can offset this deregulation, thus restoring homeostasis, possibly through a combination of cell-cell adhesion and the regulation of cell signaling. What remains to be addressed in future studies is (1) whether ectopic expression of Dsg2 alters the sensitivity of Dsg1 to degradation by ETA or loss of function upon antibody binding, and (2) whether the loss of Dsg1 function in pemphigus is caused by altered signaling and/or steric hindrance.

Currently, there are no FDA-approved prescription drugs specifically for the treatment of pemphigus. The combination of corticosteroids and other nonsteroidal immunosuppressive or anti-inflammatory drugs offers the most effective means to lower mortality while reducing long-term morbidity due to chronic systemic exposure to steroids [25]. Several ongoing clinical trials targeting cell-signaling molecules such as p38MAPK and TNF-*a* appear promising [26, 27]. As mentioned above, we recently demonstrated that Dsg2 activates multiple growth and survival pathways, including PI 3-kinase/AKT, MEKMAPK, STAT3, and NF-*κ*B. In this paper, we show that Dsg2 can limit epidermal blister formation mediated by PF antibodies. Thus, finding a drug/agent that could increase Dsg2 levels in the skin or, preferably, activate the signaling pathways downstream of Dsg2 offers a potential therapeutic treatment for this life-threatening blistering disease.

## 4. Materials and Methods

### 4.1. Histology, Immunohistochemical Staining, and Immunoblotting

For histology, skin tissues were fixed at room temperature overnight in a 10% formalin solution. Tissues were then processed for paraffin embedding, sectioned (5 *μ*m), mounted on glass slides, and stained with Hematoxylin and Eosin. 

For immunohistochemistry, mouse skin biopsies were fixed in either OCT or 10% formalin (Sigma) and were embedded in paraffin. Tissues were sectioned (5 *μ*m) as previously described in detail [6, 17]. To detect normal human Ig, the Flag tag, or Dsg1, OCT frozen sections were fixed in methanol (−20°C) for 15 minutes, permeabilized with 1% TX-100 in PBS for 5 minutes, and blocked in IF blocking buffer (5% normal goat serum, 1% BSA, and 0.02% TX-100 in PBS) for 1 hour at RT. Tissues were incubated with primary antibodies (anti-Flag (1 : 1000, Sigma) and 27B2 (1 : 10)) overnight at 4°C and secondary antibodies for 1 hour at room temperature. Antibodies were incubated in IF blocking buffer. For polyclonal antibodies against mouse Dsg1 isoforms, paraffin embedded tissues were deparaffinized in 100% xylene (5 minutes; 3 times), 100% ethanol (5 minutes; 2 times), 95% ethanol (5 minutes; 2 times), 75% ethanol (2 minutes), 50% ethanol (2 minutes), and H_2_O (2 minutes). Antigens were retrieved in an antigen-retrieving medium (Signet, Dedham, MA) by the microwave method and digestion with trypsin (Sigma). Primary and secondary antibodies were suspended in IF blocking buffer. Nuclei were stained with DAPI (Sigma), and slides were mounted for analysis via fluorescence microscopy. Polyclonal antibodies against mouse Dsg1 include AP61 (Dsg1-*α*; 1 : 100), AP498 (Dsg1-*β*, 1 : 100), and Ab15 (Dsg1-*γ*, 1 : 1000). Alexa Fluor-conjugated secondary antibodies (488 and 594 nm) were from Invitrogen (Eugene, OR) and were used at 1 : 200. Images were acquired using a Hamamatsu monochromatic digital camera and Phase 3 Imaging Systems software (Glen Mills, PA, USA; C4742–95). 

In preparation for immunoblotting, mouse back skin was pulverized in liquid nitrogen in RIPA buffer (50 mM Tris-HCl (pH 7.5), 150 mM NaCl, 1% Nonidet P-40, 0.5% deoxycholate, 0.1% SDS, and a protease inhibitor cocktail (Roche Diagnostics, Indianapolis, IN)). Protein concentration was determined (Pierce BCA kit, Pierce Biotech, Rockford, IL), and immunoblotting was performed, as described previously [17], with 20 *μ*g of protein in each lane resolved with 7% SDS-PAGE (Bio-Rad Laboratories, Hercules, CA). Signals were detected with chemiluminescence (ECL; Amersham Biosciences, Piscataway, NJ). Antibodies used were 27B2 (1 : 100), Flag (1 : 1000), and Actin (1 : 10 000).

### 4.2. PF Ig Purification

Preparation of serum Ig was done as previously described [28]. Briefly, human sera (10–20 ml) were dialyzed overnight at 4°C against 20 mM KH_2_P0_4_ (pH 8.0), and 0.02% sodium azide and was purified with a DEAE Affi-Gel Blue column (Bio-Rad Labs, Hercules, CA). Serum Ig was concentrated using Centriprep10 (Amicon Millipore, Billerica, MA) and was dialyzed against PBS at 4°C. Protein concentration was determined, and the sera were stored at −80°C.

### 4.3. Passive Transfer of Pemphigus Ig and Injection of ETA into Neonatal Mice

Newborn mice (~2 g) were injected subcutaneously with purified Ig (10 mg in 100 *μ*l) between the shoulder blades with a 1 cc insulin syringe (Becton Dickinson) as previously described [6]. For ETA treatment, newborn control and Tg mice were injected subcutaneously in the back of the neck with either ETA (0.5 *μ*g in 50 *μ*l PBS, Toxin Technology, Sarasota, FL) or PBS alone as previously described [8]. Animals were photographed and sacrificed, and their back skin was processed in 10% PBS-buffered formalin (Sigma) for histology and in OCT for immunohistochemistry or was frozen in liquid nitrogen for DNA and protein extraction. 

## Abbreviations

ETA: Exfoliative toxin A
Dsg: Desmoglein
PF: Pemphigus foliaceus
PV: Pemphigus vulgaris
Tg: Transgenic
WT: Wild-type.
